# Seroprevalence of seven climate-sensitive zoonoses in Greenland and northern Sweden (1998–2017): High antibody prevalence against *Rickettsia* and *Leptospira*, with *Leptospira* possibly linked to global warming

**DOI:** 10.1016/j.onehlt.2025.101244

**Published:** 2025-10-15

**Authors:** Anders Koch, Emilie Andersen-Ranberg, Bolette Søborg, Birgitta Evengård, Mikael Andersson, Lukas Frans Ocias, Christian Sonne, Rune Dietz, Eva Cecilie Bonefeld-Jørgensen, Jens Søndergaard, Karen A. Krogfelt, Charlotte Sværke Jørgensen

**Affiliations:** a Department of Infectious Disease Epidemiology and Prevention, Statens Serum Institut, Artillerivej 5, 2300 Copenhagen S, Denmark; b Department of Infectious Diseases, Rigshospitalet University Hospital, Blegdamsvej 9, 2100 Copenhagen Ø, Denmark; c Ilisimatusarfik, University of Greenland, Manutooq 1, 3905 Nuussuaq, Greenland; d Department of Internal Medicine, Queen Ingrids Hospital, Jens Kreutzmannip aqq. 11, 3900 Nuuk, Greenland; e Department of Veterinary Clinical Sciences, Faulty of Health and Medical Sciences, University of Copenhagen, Dyrlægevej 16, 1870 Frederiksberg, Denmark; f Aarhus University, Faculty of Technical Sciences (TECH), Department of Ecoscience (ECOS), Frederiksborgvej 399, PO Box 358, 4000 Roskilde, Denmark; g Department of Clinical Microbiology, Universitetstorget 4, 901 87 Umeå, Sweden; h Department of Epidemiology Research, Statens Serum Institut, Artillerivej 5, DK-2300 Copenhagen S, Denmark; i Department of Clinical Microbiology, Karlstad Hospital, Karlstad, Sweden; j School of Medical Science, Faculty of Medicine and Health, Örebro University, Örebro, Sweden; k Centre for Arctic Health & Molecular Epidemiology, Department of Public Health, Aarhus University, Bartholin's Allé 2, 8000 Aarhus, Denmark; l Department of Virology and Microbiological Preparedness, Statens Serum Institut, Artillerivej 5, 2300 Copenhagen S, Denmark; m Department of Science and Environment, Roskilde University, Universitetsvej 1, 4000 Roskilde, Denmark

**Keywords:** Arctic, Borreliosis, Brucella, Climate changes, Climate sensitive infections, Coxiella, *Francisella tularensis*, Greenland, Leptospira, Northern Scandinavia, Rickettsia, Seroprevalence, Tick-borne encephalitis, Zoonoses

## Abstract

**Background:**

Climate change may alter zoonotic disease patterns in the Arctic, yet knowledge remains limited.

**Design:**

Antibodies to seven zoonotic pathogens were analyzed in 660 unselected human sera drawn from serum banks from Greenland (*n* = 460) and Northern Sweden (*n* = 200) (1998–2017), frequency-matched with respect to sex, age, ethnicity and place of living. Greenlandic samples were tested for *Francisella tularensis, Brucella melitensis*, *Brucella abortus, Coxiella burnetii, Rickettsia* spp., and *Leptospira* spp., while Swedish samples also included *Borrelia burgdorferi* sensu lato (*Bbsl*) and tick-borne encephalitis virus (TBEV).

**Results:**

*Leptospira* seroprevalence was higher in Greenland 2013 (18 %, 95 % CI 13–24 %) than in Sweden 2012–2017 (4 %, 95 % CI 2–8 %) and increased significantly over time in West Greenland (1998: 2.5 %, 95 % CI 0.8–6 %; 2013: 30 %, 95 % CI 18–45 %, *p* < 0.001, OR 16.7, 95 % CI 5.7–48.9). *Rickettsia* seroprevalence remained stable over time (12 %, 95 % CI 5–24 %). Seroprevalence of *F. tularensis and B. melitensis/abortus* in Greenland 2013 was less than 1 %. In Sweden, seroprevalence was 1 % (95 % CI 0.1–4 %) for *B. melitensis/abortus*, 2 % (95 % CI 0.1–5 %) for *Bbsl*, 3 % (95 % CI 1–6 %) for *F. tularensis*, and 5 % (95 % CI 2–9 %) for TBEV. Antibodies to *C. burnetii* were not detected in any sample. Two of 81 polar bear samples from East Greenland (2016–2023) were seropositive for *Leptospira* spp.

**Conclusions:**

This first report on human *Leptospira* infection in Greenland highlights rising seroprevalence, possibly linked to contaminated water and global warming. Findings emphasize widespread *Rickettsia* exposure in northern regions and tick-borne pathogens in Sweden, underscoring the need for updated public health data to inform public health planning.

## Highlights

• Baseline seroprevalence of 7 zoonoses in humans from Greenland & N. Sweden, 1998–2017.
• Most of these microorganisms addressed for the first time in Greenland.
• *Leptospira* seropositivity seen for first time in Greenland; rates rising over time.
• Rickettsia exposure is widespread in Greenland and Northern Sweden.
• Tick-borne pathogen exposure is high in Northern Sweden.

## 1 Introduction

The Arctic experiences climate changes nearly four times faster than the global average, known as Arctic amplification [1]. This rapid warming affects ecosystems, allowing new species to move northward and potentially increasing climate-sensitive infections by altering the distribution of animal hosts and vectors, as well as impacting water supply and sewage conditions [2]. Despite significant climate changes, there is limited knowledge about climate-sensitive infections in northern regions, particularly in Greenland [3]. A review of nine climate-sensitive human diseases (anthrax, Lyme borreliosis, brucellosis, cryptosporidiosis, leptospirosis, nephropathia epidemica, Q fever, tick-borne encephalitis, and tularemia) revealed that only one human case of Q fever was reported in Greenland between 1965 and 2016 [4,5]. Greenland's limited laboratory facilities hinder the accuracy of national disease data. Despite Sweden has better laboratory resources, knowledge about these infections in its northern regions likewise remains limited.

To assess the effects of climate change on relevant infectious diseases in Arctic areas, baseline exposure to such zoonosis-causing agents in those areas needs to be estimated. In this study, we aimed to determine the prevalence of antibodies to five climate-sensitive, zoonosis-causing agents in serum samples from middle-aged Greenlanders in 2013–2015 and Swedes from Northern Sweden (Umeå community, 63.8° N lat.) 2012–2017. The study moreover tested whether the prevalence of antibodies to these agents had increased over time in Greenland by comparing samples from 1998 to 2013.

The agents studied were *Brucella melitensis* and *Brucella abortus* (brucellosis), *Leptospira* species (leptospirosis), *Coxiella burnetii* (Q fever), *Rickettsia* species (rickettsiosis), and *Francisella tularensis* (tularemia). In Sweden, *Borrelia burgdorferi* sensu lato (*Bbsl*; Lyme borreliosis) and tick-borne encephalitis virus (TBEV) were also assessed. This study was carried out as a part of the NordForsk supported CLINF project (‘Climate-change effects on the epidemiology of infectious diseases and the impacts on Northern societies’) and the AMAP (Arctic Monitoring and Assessment Programme).

## 2 Materials and methods

### 2.1 Materials

**Fig. 1:**
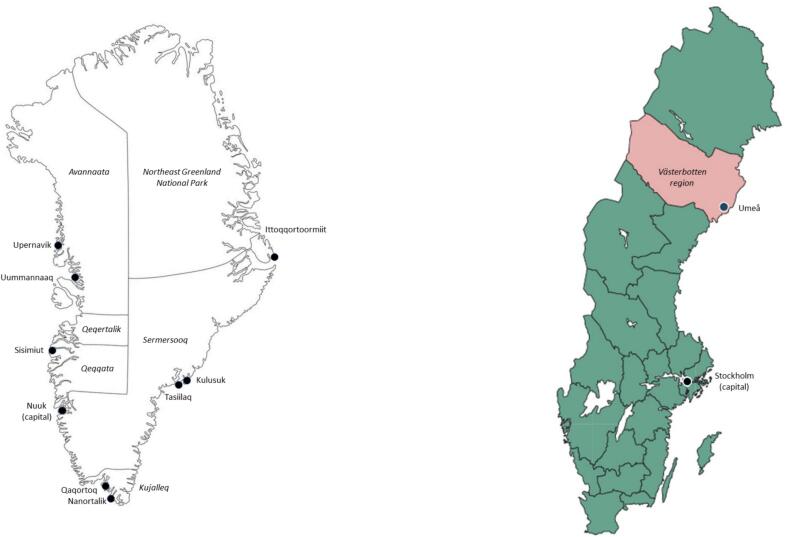
Map of Greenland and Sweden with study locations for blood sampling indicated.

The study used stored human serum samples from two Arctic regions, Greenland and the Umeå community in Västerbotten Län (region) in Northern Sweden (Fig. 1). The population of Greenland was 56,370 by January 2013 and 56,699 by January 1, 2024, 90 % Inuit (ethnic Greenlanders) (Statistics Greenland, assessed 2025). The population of Umeå community was 118,349 by the year 2013 and 133,091 by 2023; the population of Västerbotten 261,112 by 2013 and 278,729 by 2023 (Statistics Sweden, assessed 2025).

A total of 660 serum samples from four studies originally set up for other purposes were used (Table 1):

1. **INGO study (2013)** [6]: 1570 unselected Greenlanders aged 18–55 were sampled in 2013 for metabolic markers and associated genetic variants. From this group, 200 serum samples (selection criteria: age + 40 years; 25 % from each of the four regions of Greenland (in total six towns); all ethnic Greenlanders/Inuit defined as themselves and parents being born in Greenland) were randomly chosen. (INGO = Inuit Genome study)
2. **AMAP study, East Greenland (2013–2015)** [7]: Serum samples from a group of 60 persons drawn 2013–2015 (mean age 40, range 18–61, 67 % males) from the settlement Kulusuk and the towns of Tasiilaq and Ittoqqortoormiit. (AMAP = Arctic Monitoring and Assessment Programme)
3. **Sisimiut, West Greenland (1998)** [8]: 2858 unselected residents from the town of Sisimiut were screened in 1998 for a number of infectious diseases. From this group, 200 serum samples (selection criteria: age 40–50 years; 50 % males; all being Inuit) were randomly chosen.
4. **Umeå, Northern Sweden (2012–2017)** [9]: Of serum samples drawn 2012–2017 from individuals participating in the Västerbotten Intervention Programme we chose 200 sera (selection criteria: age 49–50 years; 50 % males; 50 % living in Umeå town and 50 % living outside of Umeå town in the Umeå municipality.

**Table 1:** Demographics of persons from Greenland and Northern Sweden with stored blood samples sampled 1998–2017 and tested for climate sensitive infections.

		***n***	**Mean age (years)**	**Age range** **(years)**	**Sex** **(% females)**
**Greenland 2013 (INGO study**^a^**)**					
Total		200	46.5	40–68	61 %
North	Total	50	45.5	40–56	
	Upernavik	20	45.3	40–51	75 %
	Uummannaaq	30	45.6	40–54	53 %
West	Sisimiut	50	45.9	40–68	70 %
South	Total	50	47.5	40–68	46 %
	Qaqortoq	21	49.0	40–68	33 %
	Nanortalik	29	46.4	40–53	55 %
East	Tasiilaq	50	47.0	41–56	66 %

**East Greenland 2013–15** **(AMAP study**^b^**)**					
Total^c^		60	39.3	18-61	33 %
	Tasiilaq	19	35.9	21–61	26 %
	Kulusuk	16	36.2	18–59	19 %
	Ittoqqortoormiit^c^	25	44.2	20-60	48 %

**Greenland 1998**					
West	Sisimiut	200	45.8	42–49	50 %

**Northern Sweden 2012–17**					
	Umeå Town	100	50	49–50	50 %
	Umeå County	100	50	49–50	50 %

Years indicate time of sampling.

a INGO: Inuit Genome Study.

b AMAP: Arctic Monitoring and Assessment programme.

c Information of age and sex for two persons missing, omitted from calculations including age and sex.

Except for information on sex and age (all collections), ethnicity in studies 1–3 and occupation in study 2 (East Greenlanders 2013–2015), no other information was available for study persons relevant to this study. Samples were stored frozen at −20 or -80^o^ C, thawed and analyzed anonymously. To compare with the human serological analyses, 81 plasma samples from polar bears (*Ursus maritimus*), collected between 2016 and 2023 were selected from samples collected annually in the vicinity of Ittoqqortoormiit in Northeast Greenland. These samples were thawed and tested for *Leptospira* antibodies using the same laboratory methodology as for human samples.

### 2.2 Serological analyses

Serum samples were analyzed at Statens Serum Institut, Copenhagen, Denmark, for antibodies to *B. melitensis/abortus* (tube agglutination, Reagensia AB, Denmark)*, C. burnetii* (IgG and IgM, indirect immunofluorescence assay, Focus Diagnostics, USA)*, F. tularensis* (tube agglutination)*, Leptospira* spp. (17 serovars, microagglutination test (MAT)), and *Rickettsia* spp. (IgG and IgM, indirect immunofluorescence assay, Focus Diagnostics, USA). In addition, samples from Northern Sweden (Umeå) were tested for antibodies to *Bbsl* (IgG and IgM, in-house indirect ELISA) and TBEV (Enzygnost Anti-TBE virus (IgG, IgM) ELISA). Full technical details on serological analyses are described in Supplementary material.

### 2.3 Statistical analyses

Statistical analyses were conducted using the Stats package in R, version 2022.07.0 Build 548. 95 % confidence intervals were calculated around proportions. Differences in seroprevalence proportions were tested using Chi-square test, or Fisher's exact test if expected number in any cell was below five. *p*-values <0.05 were considered statistically significant. Logistic regression analysis was used to estimate Odds rations for seropositivity to compared microbiological agents in samples from 1998 to 2013 in Greenland. Except for sex and exact age of two persons and occupations of seven persons from the AMAP study East Greenland samples (2013–2015), there were no missing data in the data sets. The two persons with missing information of sex and age were omitted from calculations of these factors, while the seven persons of unknown occupations were grouped with other occupations than hunters in the calculations of seroprevalence in hunters/non-hunters. The selection procedure for samples for our study from the original serum banks was made to construct comparable groups with respect to sex, age and region, which did not allow for control of possible confounding.

### 2.4 Ethical considerations

The study was approved by the Scientific Ethics Committee of Greenland (ref. no 2018–21077) and was reported to the Danish Data Protection Agency. Original studies from Greenland were also ethically approved (refs. KVUG J. no. 505–46, Nunatsinni Nakorsaaneqarfik J. no. 2013–084245, and J. no. 2016–19,397). Participants from both Greenland and Sweden provided written informed consent for storage and future use of their samples. The study adhered to the Helsinki II declaration and did not involve any study participants.

## 3 Results

Table 1 shows the demographics of study participants, with samples from Greenland (Sisimiut 1998 and INGO study 2013 samples, according to Materials) and Northern Sweden (Umeå, 2012–2017 samples) selected by age, sex, and region. The AMAP East Greenland study (2013–2015) samples were drawn by region only, leading to more variation in sex and age. Table 2, Table 3, Table 4 present serological results. In Greenland, antibodies to all agents except *C. burnetii* were detected (Table 2). In the 2013 Greenland INGO study samples, antibodies to *Leptospira* spp. (18 %) and *Rickettsia* spp. (5 %) were most prevalent, while antibodies to *B. melitensis/abortus* and *F. tularensis* were rare (0.5 %). Total *Leptospira* antibodies were more common in male than female samples (24 % vs. 14 %, *p* = 0.06), and higher in samples from West and South Greenland than North and East. Among persons with detectable antibodies to *Rickettsia* spp., 79 % were directed against the spotted fever group (SFG) and 29 % against the typhus group (TG) (Table 2 and Supplementary Fig. 1).

**Table 2:** Human seropositivity against five zoonotic agents in three collections of human sera from Greenland from 1998, 2013 and 2013–15^d^.

**Agent**	**Total**	**Sex**	***p*-value** **sex**	**Region**				***p*-value regions**
	*n* (%; 95 % CI)	Male/Female (% of each sex; 95 % CI)		North (%; 95 % CI	West (%; 95 % CI	South (%; 95 % CI	East (%; 95 % CI	
Greenland 2013 (INGO study, *n* = 200)		*n* = 78/122		*n* = 50	*n* = 50	*n* = 50	*n* = 50	
*Brucella* spp.	1 (0.5 %; 0.01–2.8 %)^a^	1 (1.3 %; 0–7 %)/ 0 (0 %; 0–3 %)	0.39^d^	0 (0 %; 0–7 %)	0 (0 %; 0–7 %)	1 (2 %; 0–11 %	0 (0 %; 0–7 %)	–
*Coxiella burnettii*	0 (0 %; 0–2 %)	0 (0 %; 0–5 %)/ 0 (0 %; 0–3 %)		0 (0 %; 0–7 %)	0 (0 %; 0–7 %)	0 (0 %; 0–7 %)	0 (0 %; 0–7 %)	–
*Francisella tularensis*	1 (0.5 %; 0.01–2.8 %)	1 (1.3 %; 0–7 %)/ 0 (0 %; 0–3 %)	0.39^d^	1 (2 %; 0–11 %)	0 (0 %; 0–7 %)	0 (0 %; 0–7 %)	0 (0 %; 0–7 %)	–
*Leptospira* spp.	36 (18 %; 13–24 %)	19 (24 %; 15–35 %)/ 17 (14 %; 8–21 %))	0.09	3 (6 %; 1–17 %)	15 (30 %; 18–45 %)	10 (20 %; 10–34 %)	8 (16 %; 7–29 %)	0.02
*Rickettsia* spp.	14 (7 %; 4–11 %)	5 (6 %; 2–14 %)/ 9 (7 %; 3–14 %)	1	1 (2 %; 0–11 %)	6 (12 %; 5–24 %)	6 (12 %; 5–24 %)	1 (2 %; 0–11 %)	0.04^d^
*Rickettsia* spp. Spotted fever group (SFG)	11 (6 %; 3–10 %)	5 (6 %; 2–14 %)/ 6 (5 %; 2–10 %)	0.75^d^	1 (2 %; 0–11 %)	5 (10 %; 3–22 %)	4 (8 %; 2–19 %)	1 (2 %; 0–11 %)	0.26^d^
*Rickettsia* spp. Typhus group (TG)	4 (2 %; 0.5–5 %)	0 (0 %; 0–5 %)/ 4 (3 %; 1–8 %)	0.16^d^	0 (0 %; 0–7 %)	1 (2 %; 0–11 %)	2 (4 %; 0.5–14 %)	1 (2 %; 0–11 %)	0.9^d^
Both *Rickettsia* spp. SFG and TG	1 (0.5 %; 0.01–2.8 %)	0 (0 %; 0–5 %)/ 1 (0.8 %; 0–4 %)		0 (0 %; 0–7 %)	0 (0 %; 0–7 %)	0 (0 %; 0–7 %)	1 (2 %; 0–11 %)	–
East Greenland 2013–15 (AMAP study, *n* = 60)		*N* = 39/19^b^						
*Brucella* spp.	2 (3.3 %; 0.4–12 %)^c^			–	–	–	2 (3.3 %; 0.4–12 %)^c^	NA
*Coxiella burnettii*	0 (0 %; 0–6 %)	0 (0 %; 0–9 %)/ 0 (0 %; 0–18 %)		–	–	–	0 (0 %; 0–6 %)	NA
*Francisella tularensis*	1 (1.7 %; 0.04–9 %)	0 (0 %; 0–9 %)/ 1 (5 %; 0.1–26 %)	0.33^d^	–	–	–	1 (1.7 %; 0.04–9 %)	NA
Leptospira spp.	18 (30 %; 19–43 %)	11 (28 %; 15–44 %)/ 7 (37 %; 16–62 %)	0.7	–	–	–	18 (30 %; 19–43 %)	NA
*Rickettsia* spp.	8 (13 %; 6–25 %)	7 (18 %; 8–34 %)/ 1 (5 %; 0.1–26 %)	0.25^d^	–	–	–	8 (13 %; 6–25 %)	NA
*Rickettsia* spp. spotted fever group (SFG)	8 (13 %; 6–25 %)	7 (18 %; 8–34 %)/ 1 (5 %; 0.1–26 %)	0.25^d^	–	–	–	8 (13 %; 6–25 %)	NA
*Rickettsia* spp. typhus group (TG)	3 (5 %; 1–14 %)	3 (8 %; 2–21 %)/ 0 (0 %; 0–18 %)	0.54^d^	–	–	–	3 (5 %; 1–14 %)	NA
Both *Rickettsia* spp. SFG and TG	3 (5 %, 1–14 %)	3 (8 %; 2–21 %)/ 0 (0 %; 0–18 %)					3 (5 %; 1–14 %)	NA
Greenland 1998 (*n* = 200)		100/100						
*Leptospira* spp.	5 (2.5 %; 0.8–6 %)	4 (4 %; 1–10 %)/ 1 (1 %; 0.03–5 %)	0.37^d^	–	5 (2.5 %; 0.8–6 %)	–	–	NA
*Rickettsia* spp.	25 (12.5 %; 8–18 %)	6 (6 %; 2–13 %)/ 19 (19 %; 12–28 %)	0.01		25 (12.5 %; 8–18 %)			NA
*Rickettsia* spp. spotted fever group (SFG)	19 (9.5 %; 6–14 %)	5 (5 %; 2–11 %)/ 14 (14 %; 8–22 %)	0.05	–	19 (9.5 %; 6–14 %)	–	–	NA
*Rickettsia* spp. typhus group (TG)	8 (4 %; 2–8 %)	2 (2 %; 0.2–7 %)/ 6 (6 %; 2–13 %)	0.28^d^		8 (4 %; 2–8 %)			NA
Both *Rickettsia* spp. SFG and TG	2 (1 %; 0.1–4 %)	1 (1 %; 0.03–5 %)/ 1 (1 %; 0.03–5 %)	–		2 (1 %; 0.1–4 %)			NA

*p*-values Chi-squared test or Fisher's exact test.

a Seropositive for *B. abortus*, seronegative for *B. melitensis.*

b Sex unknown for two individuals.

c Both samples seropositive for *B. abortus* and *B. melitensis.*

d Fisher's exact test.

**Table 3:** Seropositivity against five zoonotic agents by occupation in human sera from East Greenland sampled 2013–15 (*n* = 60, AMAP study)^b^.

**Agent**	**All *n*** **=** **60**			**Males only *n*** **=** **39**		
	Hunters *n* *=* 19	Other occupations/unknown^a^ *n* *=* 41	*p*-value	Hunters *n* *=* 18	Other occupations/unknown^a^ *n* *=* 21	*p*-value
	*n* (%; 95 % CI)	*n* (%; 95 % CI)		*n* (%, 95 % CI)	*n* (%; 95 % CI)	
*Brucella* spp.	0	2 (5 %; 0.6–17 %)	1^b^	0	0	NA
*Coxiella burnettii*	0	0	NA	0	0	NA
*Francisella tularensis*	0	1 (2 %; 0–13 %)	1^b^	0	0	NA
*Leptospira* spp.	5 (26 %; 9–51 %)	13 (32 %; 18–48 %)	0.9	5 (28 %; 10–53 %)	6 (29 %; 11–52 %)	1
*Rickettsia* spp.	6 (32 %; 13–57 %)	2 (5 %; 0.6–17 %)	0.01^b^	5 (28 %; 10–53 %)	2 (10 %; 1–30 %)	0.22^b^
*Rickettsia* spp. spotted fever group (SFG)	6 (32 %; 13–57 %)	2 (5 %; 0.6–17 %)	0.01^b^	5 (28 %; 10–53 %)	2 (10 %; 1–13 %)	0.22^b^
*Rickettsia* spp. typhus group (TG)	2 (11 %; 1–33 %)	1 (2 %; 0–13 %)	0.23^b^	2 (11 %; 1–35 %)	1 (5 %; 0.1–24 %)	0.6^b^
Both *Rickettsia* spp. SFG and TG	2 (11 %; 1–33 %)	1 (2 %; 0–13 %)	0.23^b^	2 (11 %; 1–35 %)	1 (5 %; 0.1–24 %)	0.6^b^

*p*-values Chi-squared test or Fisher's exact test.

a Seven persons of unknown occupation.

b Fisher's Exact test.

**Table 4:** Human seropositivity against seven zoonotic agents in human sera from Northern Sweden 2012–17 (*n* = 200).

**Agent**	**Total *n*** **(%; 95 % CI)** ***n*** **=** **200**	**Male/female** **(% of each sex)** ***n*** **=** **100/100**	***p*-value sex**	**Umeå town *n*** **(%; 95 % CI)** ***n*** **=** **100**	**Umeå municipality *n*** **(%; 95 % CI)** ***n*** **=** **100**	***p*-value location**
*Borrelia burgdorferi* sensu lato (*Bbsl)*	4 (2 %; 0.1–5 %)	0/4	0.12*	3 (3 %; 0.6–9 %)	1 (1 %; 0–5 %)	0.62*
*Brucella melitensis/abortus*	2 (1 %; 0.1–4 %)	2/0	0.5*	1 (1 %; 0–5 %)	1 (1 %; 0–5 %)	1*
*Coxiella burnettii*	0	0/0	NA	0	0	1*
*Francisella tularensis*	6 (3 %; 1–6 %)	5/1	0.21*	2 (2 %; 0.2–7 %)	4 (4 %; 1–10 %)	0.68*
*Leptospira* spp.	8 (4 %; 2–8 %)	6/2	0.28*	4 (4 %; 1–10 %)	4 (4 %; 1–10 %)	1*
*Rickettsia* spp.	23 (11.5 %; 7–17 %)	11/12	1.0	13 (13 %; 7–21 %)	10 (10 %; 5–18 %)	0.66
*Rickettsia* spp. spotted fever group (SFG)	18 (9 %; 5–14 %)	9/9	1.0	10 (10 %; 5–18 %)	8 (8 %; 4–15 %)	0.8
*Rickettsia* spp. typhus group (TG)	6 (3 %; 1–6 %)	2/4	0.68*	4 (4 %; 1–10 %)	2 (2 %; 0.2–7 %)	0.68*
Both *Rickettsia* spp. SFG and TG	1 (0.5 %; 0–3 %)	0/1 (1 %)	1*	1 (1 %; 0–5 %)	0	1*
Tick borne encephalitis virus (TBEV)	10 (5 %; 2–9 %)	2/8	0.1	5 (5 %; 2–11 %)	5 (5 %; 2–11 %)	1

*p*-values Chi-squared test or Fisher's test (*). 95 % CI in brackets.

The prevalence of *Leptospira* antibodies was higher in the West Greenland samples from 2013 (30 %) than in the West Greenland samples from 1998 (*p* < 0.001; OR 16.7, 95 % CI 5.7–48.9), while the seroprevalence of *Rickettsia* antibodies was similar (1998: 12.5 %; 2013: 12 %, *p* = 0.9; OR 0.95, 95 % CI 0.37–2.5) (Table 2). Comparing hunters to non-hunters in the East Greenland 2013–2015 samples (Table 3), the seroprevalence of *Rickettsia*, but not *Leptospira*, antibodies was higher in hunters vs. non-hunters. In the Northern Sweden samples, antibodies to all zoonotic agents except *C. burnetii* were present (Table 4). Antibodies to *Rickettsia* spp. were most prevalent (11.5 %), followed by antibodies to TBEV (5 %), *Leptospira* spp. (4 %), *F. tularensis* (3 %), *Bbsl* (2 %), and *B. melitensis/abortus* (1 %). There were no significant differences by sex nor location, except for a trend of higher *Leptospira* and *F. tularensis* exposure in males.

Among samples from Greenland with detectable antibodies to *Leptospira* spp., the serovars Icterohaemorrhagiae and Copenhageni were most frequently detected. However, reactivity against 17 and 12 serovars could be observed in serum samples from 2013 to 2015 and 1998 (Supplementary Table 1 and Supplementary Fig. 2), respectively. In the Northern Sweden samples 2012–2017, reactivity against seven *Leptospira* serovars could be observed. Of the 81 serum samples from polar bears, two displayed reactivity against *Leptospira* spp. indicating exposure to rodents. Titers were rather low and against a number of serovars (Icterohaemorrhagiae, Copenhageni, and Javanica (sample 1) and Bataviae, Tarassovi, Ballum, Cynopteri, Bratislava, Hebdomadis (sample 2)).

## 4 Discussion

This study examined serologic evidence of five climate-sensitive zoonotic infections in Greenland and seven in Northern Sweden, each transmitted through different routes. To our knowledge, the seroprevalence of some of these zoonosis-causing agents have not previously been estimated in these regions, making this study a baseline for future research. We selected samples from middle-aged individuals (mean age 50 years) with equal sex representation from both Arctic regions to ensure they had sufficient time to be exposed to these agents, if present in the environment. Additionally, we included samples from both rural and less rural areas of Greenland to obtain a representative picture of seroprevalence in both Greenland and Sweden. We found serologic evidence of all zoonoses, except of Q-fever, in humans from the two regions.

### 4.1 Leptospirosis and rickettsiosis

Antibodies to *Rickettsia* spp. and *Leptospira* spp. were prevalent in Greenland and Northern Sweden. *Rickettsia* seroprevalence was similar between countries and stable over time (1998–2013), while *Leptospira* seroprevalence was significantly higher in Greenland overall (2013–15) compared to Northern Sweden (2012–17) and West Greenland (1998). Leptospirosis is a zoonotic disease transmitted to humans, often via rodents (e.g., rats and mice) through direct contact or urine-contaminated water. Outbreaks are most common in tropical and subtropical regions during monsoons or extreme weather events involving increased water levels and water runoff. Occupational risks include abattoir workers and water sports participants. Nevertheless, leptospirosis is identified as a worldwide neglected and climate-sensitive infection [10]. Human leptospirosis is rare in Europe, with regional hot spots and potentially severe outcomes, including renal issues leading to multiorgan failure if untreated. In Denmark, the incidence is 0.23 per 100,000 (2012−23) [11].

Leptospirosis has traditionally been associated with warm, humid climates, leading to limited research in the Arctic. However, *Leptospira interrogans* has been detected in Alaskan wildlife and in ringed seals (*Pusa hispida*) in Nunavut, Canada [12]. High seropositivity (e.g., 23 % of 267 human individuals) has been reported in Canadian Indigenous communities [13], and up to 30 % seroprevalence has been observed in Yakutia, northern Russia, despite rare clinical cases [14]. This is comparable to seroprevalence values in tropical endemic regions, which can also reach 30 % [15]. No clinical cases of leptospirosis have been reported in Greenlanders to date [4]. However, the 18 % seroprevalence in the 2013 samples from four regions of Greenland supported by the Arctic studies quoted above suggests it is underdiagnosed, with many cases likely subclinical or misdiagnosed due to flu-like or nonspecific symptoms. Antibodies to serovars that were most often detected, i.e. Icterohemorhagiae and Copenhageni, indicate transmission to humans through contaminated water and/or handling of raw meat / freshwater fish, since these two serovars are most often found in rodents that can infect animals or pollute fresh water. We can only speculate about the possible causes of the high *Leptospira* spp. prevalence in Greenland. Transmission could be linked to contaminated rivers and drinking water, perhaps influenced by the release of pathogen reservoirs from melting ice and permafrost, from changes in housing conditions including sewage and water supply, or from consumption of raw ringed seal meat or potentially other marine mammals [16,17]. Given the known cross-reactivity at low titers, the multi-serovar *Leptospira* titers in the two positive polar bear samples most likely represent past exposure of undetermined origin.

In the Swedish samples, *Leptospira* seroprevalence (4 %) was significantly lower than in the Greenlandic samples. While many clinical leptospirosis cases occurred in the 1940s, fewer than 10 cases were reported annually since 1948 [18]. Between 2015 and 2024, 25 cases were reported in Sweden, with only 5 acquired domestically [19]. Improved household conditions and rat control likely contributed to this decline. However, the 4 % seroprevalence suggests leptospirosis remains underdiagnosed in Sweden and that further epidemiological surveillance is warranted in the country. Many factors could explain the difference in seroprevalence between Sweden and Greenland including different exposure to wildlife (rodents, wild boar or beavers vs. seals and other marine mammals); differences in environmental exposure with farming, fishing, forest work, and freshwater recreation being common in Northern Sweden; differences in public health structure (water treatment systems, sewage disposals, and housing isolation); and differences in cultural dietary practices including intake of raw or minimally cooked meat or fish.

Leptospirosis is climate-sensitive, with rising temperatures and precipitation driving increased incidence. Human displacement and impaired sewage systems linked to climate change may also elevate the risk [20]. In West Greenland, seropositivity rose from 2.5 % in 1998 to 30 % in 2013, alongside a temperature increase and higher precipitation 1991–2019 [21]. Similarly, EU countries saw a 5 % annual rise in cases from 2010 to 2021 [22]. In contrast, northern Scandinavia and Russia experienced a decline (1999–2015), inversely correlated with rising spring temperatures [16]. Differences in housing, sewage systems, and sampling methods, as well as the distinction between seropositivity and clinical disease and the assessment of seroprevalence in different groups at two distinct times, may explain these variations. There may be many explanations for the apparent increase in seroprevalence in West Greenland from 1998 to 2013, including, among others, increased exposure to reservoir hosts (e.g. rodent population dynamics), changes in local behavioral or dietary practices (e.g. handling or consumption of raw seal meat or fish), changes in water sanitation and housing infrastructure, and changes in occupational or subsistence-related risk factors in the involved community. Except for high housing construction activity over the past decades in many Greenlandic towns, we are not aware of other changes in possible risk factors for *Leptospira* exposure in the country. Climate changes may play a role for changes in human *Leptospira* exposure in Greenland, but from the present data, inferring causality between changes in climate and seroprevalence remains speculative and needs to be addressed in future studies.

Reactivity against all 17 serovars was detected in the Greenlandic INGO study samples from 2013, suggesting no single primary source of transmission. In contrast, reactivity against only seven serovars were observed in the Swedish samples, which may indicate fewer main reservoirs of disease in Sweden or simply reflect the lower seroprevalence observed there.

Our data do not allow us to identify the transmission routes or vectors for leptospirosis in Greenland. The detection of seropositivity in polar bears represent top of the food chain, suggests the presence of *Leptospira* in wildlife. However, in the 2013 INGO samples, the highest human seroprevalence was observed in samples from Sisimiut, a non-major hunting district, and no difference in seroprevalence was found between hunters and non-hunters in the AMAP East Greenland samples. Likewise, 60 sled dogs from east and west Greenland were recently found seronegative for *Leptospira* spp. (Ranberg-Andersen, E. unpublished data). Sled dogs are typically fed wildlife meat and hydrated by creek water from the local communal water supply, but although the predominant *Leptospira* serovars found in Greenlanders Copenhageni and Icterohaemorrhagica are known to readily infect canines, these dogs were found seronegative. This suggests that factors other than exposure to wildlife tissues may be involved in transmission. The increase in seroprevalence from 1998 to 2013 could be associated with changing housing and sewage conditions in towns.

*Rickettsia* are intracellular bacteria causing symptoms like fever, rash, and headache. They are transmitted by blood-feeding arthropods, including ticks, fleas, lice, mites, and possibly mosquitoes [23]. *Rickettsia* are divided into four groups: SFG, TG, transitional, and ancestral groups. Most *Rickettsia* belong to the SFG, primarily tick-borne species. The TG includes *Rickettsia prowazekii* and *Rickettsia typhi*, transmitted by human body lice and rat fleas, respectively, though other organisms, such as leeches and non-blood-feeding arthropods, may serve as potential vectors [24]. *Rickettsia* have been found on all continents except Antarctica, although most species are region-specific due to climatic conditions and the limitations of their vectors and natural hosts [23]. However, *R. typhi* (TG) and *R. felis* (SFG) are exceptions, occurring worldwide [23]. These species are transmitted by fleas, unlike most *Rickettsia*, which require tick vectors, thereby limiting their spread to the ticks' geographic range [23].

Despite an average seroprevalence of 7–13 %, there were no significant differences in the seroprevalence of rickettsiosis between Sweden and Greenland, nor across time and regions in Greenland from 1998 to 2015. Most seropositive cases in both regions belonged to the spotted fever group, transmitted by ticks, fleas, or mosquitoes. So far, no human cases of rickettsiosis have been reported in Greenland, suggesting it may be underdiagnosed. While ticks are unlikely vectors due to the absence of known viable tick colonies in the country, abundant mosquitoes could potentially transmit the infection [25]. Although seroprevalence was slightly higher among hunters in the East Greenland AMAP samples (2013–15), no significant differences were found across Greenlandic regions in the 2013 INGO samples, indicating hunting is not a major risk factor for rickettsiosis. While rickettsioses might be climate-sensitive infections, we found that seroprevalence in West Greenland remained unchanged over 15 years.

Although *Rickettsia* spp. have been detected in ticks in Sweden, the incidence of rickettsiosis remains unknown. In Scandinavia, the castor bean tick (*Ixodes ricinus*) is the main reservoir for tick-borne *Rickettsia*. Its distribution, influenced by temperature, altitude, humidity, and large hosts like roe deer, expanded northward in Sweden during the 1980s–1990s due to warmer climates and roe deer population growth [26]. Exposure to *Rickettsia* in Northern Sweden likely occurs through ticks, but other arthropods or non-tick reservoirs, such as birds and rodents, may also play a role.

### 4.2 *Coxiella burnetii*, *Brucella* spp. and *Francisella tularensis*

Slightly surprising, none of our samples tested seropositive for *C. burnetii* antibodies, suggesting no widespread prevalence in Greenland or northern Scandinavia, despite the first human Q fever case in the Arctic diagnosed in East Greenland in 2007 [5].

Low serologic evidence of brucellosis (*B. melitensis* and *B. abortus*) was found in the samples from Greenland and Northern Sweden. Globally, *Brucella* reservoirs include cattle, dogs, sheep, goats, pigs, and marine mammals like seals and walruses [27]. To date, thirteen species within the genus *Brucella* have been recognized, among which *B. melitensis*, *B. abortus*, *B. suis*, and *B. canis* are recognized as the most significant species because of their ability to traverse host species barriers, establish persistent infections in animal reservoirs, and elicit chronic, debilitating disease in humans [28]. However, a species like *B. canis* is clearly an underestimated zoonotic pathogen in Europe [28]. Some seals in the North Atlantic Ocean have been found seropositive for *Brucella*, although other *Brucella* species than *B. melitensis* and *B. abortus* like *B. pinnipedialis* and *B. ceti* are predominant in seals [29]. We do not know how much our *Brucella* assays cross react with marine or other *Brucella* species than *B. melitensis* and *B. abortus*, but given the low number of seropositives and at low titers in our study, we do not think *Brucella* represents a major risk to human in Greenland or Northern Sweden.

For *F. tularensis*, 2/260 samples in Greenland and 6/200 in Northern Sweden were seropositive. Northern Sweden's 3 % positivity aligns with its rise in tularemia cases since the 1980s [30]. In Greenland, no human cases or wildlife evidence of tularemia exist [4], though the infection has been found in Alaskan wildlife [12]. While ticks are major vectors, sustainable tick colonies are unknown in Greenland except perhaps for ticks transported to the Arctic attached to long distance migratory bird species [31]. Transmission could involve flies or mosquitoes.

### 4.3 *Borrelia burgdorferi* sensu lato *(Bbsl) and TBEV*

Since ticks are the main vector for *Bbsl* and TBEV and as there are no known viable colonies of ticks in Greenland, antibodies to these microorganisms were only measured in the Swedish samples. In the region of Umeå, 2 % of individuals were seropositive for *Bbsl* and 5 % for TBEV. No significant differences were found between residents of Umeå town and those living in the surrounding municipality. Studies in Scandinavia show that the *Bbsl* seroprevalence ranges from 4 % to 24 %, depending on region, population, and diagnostic methods [32]. A Finnish survey in 2011, reported seroprevalences of 2.5 %–3.5 % in people aged 40–59, comparable to our study population [32]. In contrast, samples from 1966 to 72 showed higher values of 14 %–23 % in the same age groups, with 8 % overall seroprevalence in northern Finland [33]. While specific data from Sweden are lacking, these trends suggest that an increase in *Bbsl* antibody prevalence in Northern Sweden cannot be assumed, despite the northward spread of the tick vector *I. ricinus* [26].

TBEV spreads westward in Europe, with its vector, *I. ricinus*, expanding northward in Scandinavia [26]. Northern Sweden remains a low-incidence area, with only 15 of 3893 TBE cases (0.4 %) reported from Norrbotten and Västerbotten (2015–24) [34]. However, 5 % of our study population was seropositive for TBEV, suggesting that while clinical TBE incidence is low, the risk of exposure in Northern Sweden is still present, though lower than in the south. An important limitation is that our serological test could not distinguish between vaccine-induced antibodies and antibodies from natural infection. Therefore, we cannot determine to what extent seropositivity is due to natural infection or TBEV vaccination. A recent study found 11.3 % of blood donors in the Västerbotten region to have been TBEV vaccinated [35]. While blood donors are a selected group it cannot be ruled out that at least some of the seropositive persons in our study were seropositive because of vaccination.

### 4.4 Strengths and limitations

A key strength of this study is the unbiased sample collection from residents, with most samples randomly selected, except for the occupation-specific East Greenland samples (2013–2015). Sex and age balance ensured comparability across time and locations. All analyses used were established, commercially available serologic tests. However, there are limitations. Serology reflects past infections, not current risk, as antibodies persist for varying durations and can wane over time, meaning seronegativity doesn't exclude prior exposure. We cannot confirm if seropositive individuals were infected locally, though for Greenland, only samples from lifelong residents were included, and frequent travel abroad is less likely due to high costs. Samples from citizens from Nuuk, whose travel patterns differ with more regular travels to locations outside of Greenland, were excluded. While travel may explain some seropositivity, it is unlikely to account for the observed values of *Leptospira* and *Rickettsia* seropositivity. False positives or cross-reactivity (e.g., between *Leptospira* serovars or between *Rickettsia* subtypes) cannot be ruled out, though validated methods and adjusted cut-offs were used to improve specificity. Finally, for TBEV, as stated above, we could not distinguish between vaccine-induced and naturally acquired antibodies.

Updated data such as the present on the occurrence of zoonotic infections is essential for developing risk maps and providing relevant guidance to both the healthcare sector and the public. Identifying infectious diseases in areas where they were previously unknown alerts healthcare professionals to consider these infections during diagnosis and treatment. Additionally, tracking seroprevalence of zoonotic agents over time can contribute to assessing the impact of climate change on human health.

## 5 Conclusions

This serosurvey among four serum collections, drawn in Greenland and Northern Sweden at two time points (1998 and 2013–17), assessed the baseline seroprevalence of seven climate-sensitive zoonotic agents in largely unselected persons, mostly individuals around 45–50 years old. These agents, involving various transmission routes, had not been thoroughly studied in these regions before. We found that exposure to *Leptospira* spp. and *Rickettsia* spp. is common in both countries. *Leptospira* seroprevalence was higher in Greenland (18 %) than in Northern Sweden (4 %), while *Rickettsia* seroprevalence was more similar (7 % in Greenland and 12 % in Northern Sweden). Although *Leptospira* antibodies were found in plasma samples from polar bears indicating the bacterium in wildlife, the unexpectedly high *Leptospira* human seroprevalence in Greenland that increased markedly over time suggests other transmission pathways and may reflect the effects of accelerated Arctic climate change. Increased surveillance and awareness of this pathogen in Greenland are recommended including studies of potential sources of these diseases from marine mammals. Low seroprevalence of *B. melitensis/abortus*. and *F. tularensis* (0.5–3 %) was detected in both countries, while no samples were seropositive for *C. burnetii*. In Northern Sweden, tick-borne pathogens (*Bbsl* and TBEV) are present, correlating with an expanding tick population. Updated data on zoonotic infections are essential for creating risk maps and providing guidance to healthcare professionals and the public. These results also establish baseline data for future studies on climate-sensitive infections in northern regions.

## Funding sources

Study designing, literature search, serologic and data analyses, interpretation, and manuscript preparation were conducted within the CLINF project (‘Climate change effects on the epidemiology of infectious diseases and the impacts on Northern societies’, http://www.clinf.org), funded by 10.13039/501100004785NordForsk [Grant No. 76413]. Human blood sample collection in Greenland (INGO 2013) was supported by the Danish Research Council [Grant No. 0602-02394B]. Sample collection in Sisimiut (1998) was supported by the TUPOLAR program, the 10.13039/100008392Danish Medical Research Council, and the 10.13039/501100006258Commission for Scientific Research in Greenland. DANCEA grants MST-112-00241, MST-113-00081, RISK-PFAS (2019–8201), TIME-PFAS (2021–60,245), and ZOO-PFAS (2022–86,192) supported East Greenland fieldwork and polar bear sample analyses. Additional funding came from the EU HEurope grant 101135051 (Arcsolution) and WhaleAdapt (NFRFI). Funders had no role in the manuscript.

## Ethical approval statement

The study received approval from the Scientific Ethics Committee of Greenland (ref. no 2018–21077) and was reported to the Danish Data Protection Agency. Original studies from Greenland were also ethically approved (refs. KVUG J. no. 505–46, Nunatsinni Nakorsaaneqarfik J. no. 2013–084245, and J. no. 2016–19,397). Participants from both Greenland and Sweden who provided the samples, provided informed consent for storage and future use of their samples. The study adhered to the Helsinki II declaration. The actual use of the samples did not involve participation of any study persons, nor did results have any consequence for anyone in the study. Informed consent for the specific use of these samples in this study was therefore waived.

## Generative AI and AI-assisted technologies

During the preparation of this work the first author used ChatGPT 4.0 in order to improve readability and language. After using this tool, the first author reviewed and edited the content as needed and takes full responsibility for the content of the publication.

## Author statement

The work described has not been published previously except in the form of a preprint, an abstract, a published lecture, academic thesis or registered report.

The article is not under consideration for publication elsewhere.

The article's publication is approved by all authors and tacitly or explicitly by the responsible authorities where the work was carried out.

If accepted, the article will not be published elsewhere in the same form, in English or in any other language, including electronically, without the written consent of the copyright-holder.

## CRediT authorship contribution statement

**Anders Koch:** Writing – original draft, Visualization, Validation, Resources, Project administration, Methodology, Investigation, Funding acquisition, Formal analysis, Data curation, Conceptualization. **Emilie Andersen-Ranberg:** Writing – review & editing, Investigation, Funding acquisition, Data curation, Conceptualization. **Bolette Søborg:** Writing – review & editing, Methodology, Funding acquisition, Formal analysis, Data curation, Conceptualization. **Birgitta Evengård:** Writing – review & editing, Project administration, Investigation, Funding acquisition, Formal analysis, Data curation, Conceptualization. **Mikael Andersson:** Writing – review & editing, Software, Methodology, Investigation, Formal analysis, Conceptualization. **Lukas Frans Ocias:** Writing – review & editing, Investigation, Formal analysis. **Christian Sonne:** Writing – review & editing, Funding acquisition, Formal analysis, Data curation. **Rune Dietz:** Writing – review & editing, Funding acquisition, Formal analysis, Data curation. **Eva Cecilie Bonefeld-Jørgensen:** Writing – review & editing, Funding acquisition, Formal analysis, Data curation. **Jens Søndergaard:** Writing – review & editing, Funding acquisition, Data curation. **Karen A. Krogfelt:** Writing – review & editing, Investigation, Funding acquisition, Formal analysis, Conceptualization. **Charlotte Sværke Jørgensen:** Writing – review & editing, Investigation, Funding acquisition, Formal analysis, Conceptualization.

## Declaration of competing interest

The authors declare the following financial interests/personal relationships which may be considered as potential competing interests:

Anders Koch reports financial support for this study was provided by Nordforsk. Birgitta Evengard reports financial support for this study was provided by Nordforsk. The other authors declare that they have no known competing financial interests or personal relationships that could have appeared to influence the work reported in this paper.

## Data Availability

The authors do not have permission to share data.

## Appendix A Supplementary data

Supplementary material Image 1
