# Supplementary material for: Seroprevalence of seven climate-sensitive zoonoses in Greenland and northern Sweden (1998–2017): High antibody prevalence against Rickettsia and Leptospira, with Leptospira possibly linked to global warming
Source: One Health. 2025 Oct 15;21:101244. doi: 10.1016/j.onehlt.2025.101244 (PMC12597069; doi:10.1016/j.onehlt.2025.101244)
Supplement: Supplementary file 1 — Supplementary material [file mmc1.docx]

**Supplementary material**

'*Seroprevalence of seven climate-sensitive zoonoses in Greenland and Northern Sweden (1998–2017): High Antibody Prevalence against Rickettsia and Leptospira, with Leptospira possibly linked to global warming’*

**Description of serological analyses**

Serum samples were analyzed at Statens Serum Institut, Copenhagen, Denmark, for antibodies against *Brucella melitensis, Brucella abortus, Coxiella burnetii, Francisella tularensis, Leptospira* species (17 serovars), and *Rickettsia species*. In addition, samples from Northern Sweden were tested for antibodies against *Borrelia burgdorferi* sensu lato *(Bbsl)* and Tick-borne encephalitis virus (TBEV).

*Brucella melitensis* and *Brucella abortus:* Antibodies were determined by tube agglutination with commercially available antigens *(*Reagensia AB, Denmark).

*Coxiella burnetii:* Serum samples were analyzed for IgG and IgM antibodies against phase I and II antigens using an indirect immunofluorescence (IFA) assay (Focus Diagnostics, Inc., USA)^1^.

*Francisella tularensis:* Antibodies were determined by tube agglutination^2^.

*Leptospira* spp.: Sera were tested for specific antibodies by microagglutination test (MAT) against 17 Leptospira serovars including *L. interrogans serovar Hardjo (Hardjo), L. hurstbridge hurstbridge (BUT6), L. interrogans serovar Hebdomadis (hebdomatis), L. borgpetersenii serovar Tarassovi (perepelitsin), L. kirschneri serovar Cynopteri (3522C), L. interrogans serovar Autumnalis (akiyam), L. interrogans serovar Copenhageni (M20), L. borgpetersenii serovar Javanica (VB46), L. borgpetersenii serovar Ballum (castellon 3), L. kirschneri serovar Grippotyphosa str. Moskva (Moskva V), L. interrogans serovar Icterohaemorrhagiae (RGA), L. interrogans serovar Canicola (Hond), L. borgpetersenii serovar Sejroe (M84), L. interrogans serovar Pomona (Pomona), L. interrogans serovar Bratislava (J.B.), L. interrogans serovar Bataviae (swart), and L. biflexa serovar Patoc (Patoc), as previously described*^3^.

*Rickettsia* spp.: Serum samples were analyzed for IgG and IgM antibodies against spotted fever group (SFG) and typhus group (TG) *Rickettsia* spp. using an indirect immunofluorescence (IFA) assay (Focus Diagnostics, Inc., USA). Whole-cell *Rickettsia* *rickettsii* and *Rickettsia* *typhi* were used as antigens. Samples were titrated until end-point fluorescence in two-fold dilutions and slides were read by experienced microscopists. The test was performed according to the instruction from the manufacturer, except that the IgG cut-off value for SFG *Rickettsia* was raised from 1:64 to 1:512 to increase specificity. The latter was based on a previous seroprevalence study that showed high background levels of anti-SFG *Rickettsia* IgG among healthy Danish blood donors^4^.

*Borrelia burgdorferi* sensu lato (*Bbsl*): Sera were tested using an in-house indirect ELISA identical to the commercial Oxoid IDEIA™ *Borrelia burgdorferi* IgG and IgM assay. In the IgG assay wells are coated with purified native *B. afzelii* DK1 strain flagellum as antigen. Bound IgG is detected by peroxidase-conjugated anti-human IgG. The IgM assay is a µ-capture ELISA where the wells are coated with antibodies specific to human IgM. The Borrelia-specific antibodies of the captured IgM in the sample is detected with biotinylated native *B. afzelii* DK1 strain flagella complexed with peroxidase-conjugated streptavidin using TMB as substrate.

Tick-borne encephalitis virus (TBEV): Sera were analyzed for presence of IgG and IgM antibodies against TBEV using the Enzygnost Anti-TBE virus (IgG, IgM) ELISA.

References supplementary material

1. Kantsø B, Svendsen CB, Jorgensen CS, Krogfelt KA. Comparison of two commercially available ELISA antibody test kits for detection of human antibodies against Coxiella burnetii. *Scandinavian Journal of Infectious Diseases*. 2012;44(7):489-494. doi:10.3109/00365548.2012.664777

2. Haulrig MB, Mathiasen G, Nielsen RM, Kromann CB, Krogfelt KA, Wiese L. Two cases of tick-borne transmitted tularemia on Southern Zealand, Denmark. *APMIS*. 2020;128(1):61-64. doi:10.1111/apm.13008

3. van Alphen LB, Lemcke Kunoe A, Ceper T, et al. Trends in Human Leptospirosis in Denmark, 1980 to 2012. *Eurosurveillance*. 2015;20(4):21019. doi:10.2807/1560-7917.ES2015.20.4.21019

4. Kantsø B, Svendsen CB, Jørgensen CS, Krogfelt KA. Evaluation of serological tests for the diagnosis of rickettsiosis in Denmark. *Journal of Microbiological Methods*. 2009;76(3):285-288. doi:10.1016/j.mimet.2008.12.012

**Supplementary table 1**. Data on seropositivity against 17 Leptospira serovars in blood samples from a total of 660 persons from Greenland 1998 and 2013-15* and Northern Sweden (Umeå) 2012-17. Numbers and percentages seropositive (%).

|  | **Patoc** | **Icterohaemorrhagiae** | **Copenhageni** | **Canicola** | **Autumnalis** | **Javanica** | **Hardio** | **Sejroe** | **Pomona** | **Grippotyphosa str. Moskva** | **Bataviae** | **Tarassovi** | **Ballum** | **Cynopteri** | **Hurstbridge** | **Bratislava** | **Hebdomadis** | **Total** |
| --- | --- | --- | --- | --- | --- | --- | --- | --- | --- | --- | --- | --- | --- | --- | --- | --- | --- | --- |
| Greenland  1998  N = 200 | 1 (0.5) | 3 (1.5) | 4  (2) | 0 | 1  (0.5) | 2  (1) | 1  (0.5) | 3  (1.5) | 5  (2.5) | 2  (1) | 1  (0.5) | 0 | 2  (1) | 0 | 0 | 0 | 1  (0.5) | 5  (2.5) |
| Greenland 2013-15  N = 260* | 1  (0.4) | 23  (8.8) | 21  (8.0) | 11  (4.2) | 10  (3.8) | 9  (3.4) | 7  (2.7) | 11  (4.2) | 9  (3.4) | 15  (5.7) | 8  (3.1) | 12  (4.6) | 11  (4.2) | 8  (3.1) | 2  (0.8) | 5  (1.9) | 6  (2.3) | 54  (20.7) |
| Northern Sweden 2012-17  N = 200 | 0 | 3  (1.5) | 5  (2.5) | 3  (1.5) | 0 | 1  (0.5) | 0 | 0 | 1  (0.5) | 2  (1.0) | 0 | 0 | 1  (0.5) | 0 | 0 | 0 | 0 | 8  (4.0) |
| * INGO (2013) and AMAP (2013-15) study samples | | | | | | | | | | | | | | | | | | |

**Supplementary figure 1**. Distribution of seropositivity (percentage) against *Rickettsia rickettsia* (spotted fever group, SFG) and *Rickettsia typhi* (typhus group, TG) in human samples from Greenland 1998, 2013 and 2013-15, and Northern Sweden 2012-17. Greenland samples 2013-15 consist of INGO samples (2013) and AMAP samples (East Greenland 2013-15). Y axis percentage of samples seropositive.

**Supplementary figure 2**. Distribution of seropositivity (percentage) against *Leptospira* serovars in four serum collections sampled 1998-2017 in Greenland and Northern Sweden. Y axis percentage seropositive. Greenland samples 2013-15 consist of INGO samples (2013) and AMAP samples (East Greenland 2013-15). Y axis percentage seropositive.
